# Hepatobiliary Adverse Events Linked to Immune Checkpoint Inhibitors: A Real‐World Pharmacovigilance Analysis Using FAERS Data

**DOI:** 10.1111/1759-7714.70184

**Published:** 2025-11-16

**Authors:** Yuzhu Chen, Yixin Zeng, Kaisheng Zhang, Nand Lal, Yuxiang Zhao, Fei Qi, Tongmei Zhang

**Affiliations:** ^1^ Department of Oncology, Beijing Chest Hospital Capital Medical University, Beijing Tuberculosis and Thoracic Tumor Research Institute Beijing China; ^2^ Laboratory for Clinical Medicine Capital Medical University Beijing China; ^3^ School of Medicine Shaoguan University Shaoguan People's Republic of China; ^4^ Department of Physiology Harbin Medical University Harbin Heilongjiang China; ^5^ Department of Clinical Medicine Fenyang College of Shanxi Medical University Taiyuan Shanxi China

**Keywords:** disproportionality analysis, drug‐specific risk, FAERS, hepatobiliary toxicity, immune checkpoint inhibitors

## Abstract

**Background:**

Immune checkpoint inhibitors (ICIs) improve cancer outcomes but cause significant hepatobiliary toxicity (e.g., liver dysfunction, immune‐mediated liver disease). Prior studies were limited to small samples or case reports. This study evaluated hepatobiliary toxicity, risk variations, and temporal trends for six ICIs (PD‐1/PD‐L1 inhibitors) via the FDA Adverse Event Reporting System (FAERS) to guide safer use.

**Methods:**

We analyzed 18 640 061 FAERS reports (2004–2024). Disproportionality analyses (ROR, PRR, EBGM, BCPNN) detected hepatobiliary toxicity signals. A Weibull model analyzed AE timing. Logistic regression assessed effects of age, gender, and weight.

**Results:**

PD‐L1 inhibitors (Durvalumab, Atezolizumab) are associated more strongly with immune‐mediated liver disease/hepatic failure (ROR = 4.28–5.07). PD‐1 inhibitors (Nivolumab, Pembrolizumab) are linked more to hepatitis/liver abnormalities (ROR = 3.42–3.93). AE reports are common in males (53.32%) and patients > 65 years (43.43%), though demographics didn't alter risk (*p* > 0.05). Median onset: 23 days (Toripalimab liver injury) vs. 84 days (Tislelizumab autoimmune hepatitis), supporting drug‐specific monitoring in the first three months.

**Conclusion:**

Our FAERS analysis showed PD‐L1 inhibitors were more associated with immune‐mediated liver disease and hepatic failure, whereas PD‐1 inhibitors were linked to hepatitis and liver abnormalities, underscoring the need for drug‐specific monitoring.

## Graphical abstract

This FAERS pharmacovigilance study reveals distinct hepatotoxicity profiles between PD‐1 and PD‐L1 inhibitors. PD‐L1 inhibitors are more associated with immune‐mediated liver disease and hepatic failure, while PD‐1 inhibitors are linked to hepatitis and liver abnormalities. The median time to onset varies significantly, from 23 days (Toripalimab) to 84 days (Tislelizumab), highlighting the necessity for drug‐specific monitoring within the first 3 months of therapy.

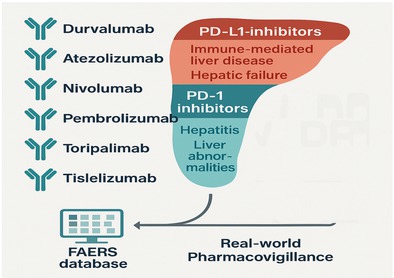

## 1 Introduction

ICIs are innovative anti‐cancer treatments that boost the immune response to tumors by inhibiting checkpoint pathways like PD‐1/PD‐L1 between tumor and immune cells. These therapies have led to unprecedented breakthroughs across various cancer types, significantly prolonging survival in some patients and bringing new hope to oncology [1]. The advent of ICIs has not only transformed traditional cancer treatment paradigms but has also reshaped our understanding of tumor immunotherapy, which is now a standard of care for numerous malignancies with expanding indications.

The widespread use of ICIs in clinical settings has resulted in the appearance of numerous immune‐related adverse events (irAEs), garnering significant attention from clinicians [2]. Among these, hepatobiliary toxicities—such as abnormal liver function, immune‐mediated liver disease, and hepatitis—are particularly concerning due to their potential to compromise treatment adherence and clinical outcomes [3]. The underlying mechanisms are not yet fully elucidated, but accumulating evidence suggests links to epitope spreading and disruption of hepatic immune tolerance. During ICI therapy, tumor cell lysis releases diverse proteins and host antigens, which, under conditions of inflammation and tissue damage, are presented by antigen‐presenting cells. This can activate autoreactive T cells and trigger liver injury [1]. The liver typically sustains immune tolerance via anti‐inflammatory signals from both its parenchymal and non‐parenchymal cells. ICIs can block these regulatory pathways, disrupt immune homeostasis, and promote liver susceptibility to pharmacologic or microbial insults, resulting in immune‐mediated liver injury [4].

Most prior studies of ICI‐related hepatobiliary toxicity have been limited to case reports or small cohorts, lacking systematic evaluation and failing to capture broader toxicity patterns. Furthermore, different ICIs, such as PD‐1 and PD‐L1 inhibitors, may exhibit distinct hepatobiliary toxicity profiles, onset patterns, and risk characteristics due to differences in their mechanisms of action. To verify this hypothesis, the present study is the first to utilize publicly available FAERS data to analyze the hepatobiliary toxicity spectrum associated with six commonly used ICIs—Durvalumab, Atezolizumab, Nivolumab, Pembrolizumab, Toripalimab, and Tislelizumab—utilizing information from the publicly available FAERS [5]. Advanced data‐mining techniques were employed to assess toxicity profiles, and the associations of hepatobiliary events with variables such as drug type, age, sex, and other factors were evaluated using disproportionality analysis. This study seeks to offer evidence‐based insights for the clinical application of ICIs, optimizing therapeutic benefits and reducing hepatobiliary risks to improve patient outcomes and quality of life.

## 2 Materials and Methods

### 2.1 Data Sources

The primary data for this study were obtained from the FAERS database (https://fis.fda.gov/extensions/FPD‐QDE‐FAERS/FPD‐QDE‐FAERS.html). FAERS is a critical pharmacovigilance system maintained and quarterly updated by the FDA. The database is distributed in compressed XML format and contains seven data files: DEMO (patient demographic information), DRUG (drug/biologic product information), REAC (encoded adverse event terms), OUTC (patient outcomes), RPSR (report source information), THER (drug therapy start and end dates), and INDI (indication codes). FAERS collects spontaneous AE reports from healthcare professionals, manufacturers, and consumers worldwide, and is widely utilized for drug safety signal detection. To remove duplicate reports, we adhered to the FDA‐recommended deduplication method: For reports sharing the same CASEID, we retained the one with the most recent FDA_DT (date of FDA receipt); when both CASEID and FDA_DT were identical, the report with the highest PRIMARYID was retained.

**FIGURE 1:**
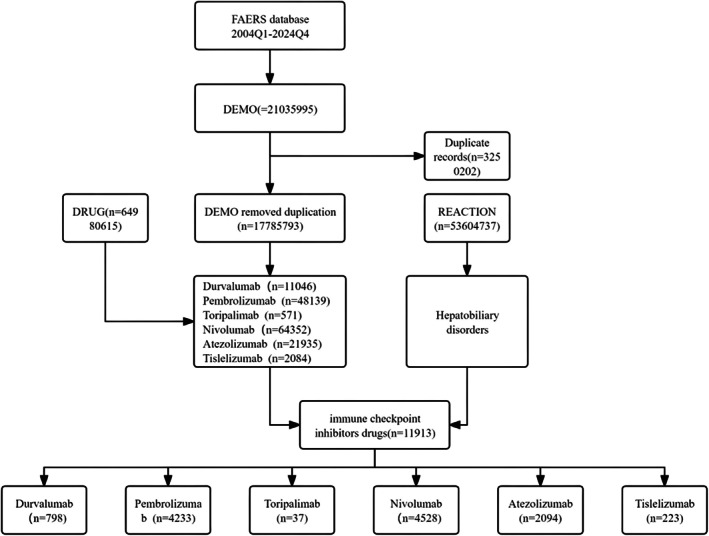
Flowchart illustrating the selection process of immune checkpoint inhibitor–containing reports from the FAERS database under the hepatobiliary system disorders classification.

This study analyzed 25 000 088 reports submitted from Q1 2004 to Q4 2024. A systematic de‐duplication strategy, based on two defined criteria, was employed to eliminate redundant entries. After de‐duplication, 18 640 061 unique reports remained for analysis. Figure 1 illustrates the de‐duplication workflow.

### 2.2 Data Extraction

We searched the FAERS database using both generic and trade names of FDA‐approved ICIs to obtain reports of associated AEs. Our analysis focused on AEs categorized under the System Organ Classification (SOC) for Hepatobiliary System Disorders (code: 10019805), allowing us to identify events specifically related to hepatobiliary toxicity.

Additionally, we examined the time until these AEs began for each ICI, defined as the interval from the start of ICI therapy to when the adverse event occurred. The analysis included only cases where the time‐to‐onset exceeded zero days. Any reports with erroneous or missing date data, including those where the start date was after the event date, were not included in the dataset.

### 2.3 Signal Mining

Analysis of disproportionality is a common technique in post‐marketing pharmacovigilance for identifying adverse drug reaction signals. This method incorporates statistical metrics like the reporting odds ratio (ROR), proportional reporting ratio (PRR), Empirical Bayesian Geometric Mean (EBGM), and Bayesian Confidence Propagation Neural Network (BCPNN). This study utilized four metrics to detect hepatobiliary adverse event signals linked to ICIs.

We compared the frequency of hepatobiliary‐related AEs reported for ICIs compared to the whole FAERS database. A disproportionality analysis was conducted, calculating RORs, where a positive signal was identified if the 95% confidence interval's (CI) lower limit exceeded 1, indicating a possible connection between the ICI and liver‐related toxicity.

To reduce potential bias and enhance the robustness of the results, this study employed a multivariate logistic regression model for sensitivity analysis to reassess the association between exposure to ICIs and the occurrence of hepatobiliary‐related AEs. Adjusted covariates were included to estimate the adjusted reporting odds ratios (aRORs) with 95% CIs. Reports with missing data on key covariates were excluded to ensure the validity of the multivariate logistic regression analysis. Additionally, the most common concomitant medications known to potentially cause hepatobiliary‐related AEs, along with other relevant variables, were incorporated as confounding factors in the logistic regression model. Additionally, subgroup analyses were conducted.

The period from the start of ICI treatment (noted in the THER file) to the appearance of an adverse event (noted in the DEMO file) was defined as the time to onset of irAEs. To identify the optimal distribution for the TTO analysis, a goodness‐of‐fit assessment was conducted using the Weibull, gamma, and exponential distributions, characterized by the parameters *α* (scale) and *β* (shape).

Furthermore, a logistic regression analysis was performed to assess age, body weight, and sex as possible risk factors for hepatobiliary AEs. Reports that were incomplete were not included, and categorical variables were examined to find significant predictors. Statistical analyses were performed using R software, version 4.4.1.

## 3 Results

### 3.1 Descriptive Analysis

Hepatobiliary system disorder‐related AEs were assessed for the ICIs: Durvalumab (*n* = 798), Pembrolizumab (*n* = 4233), Toripalimab (*n* = 37), Nivolumab (*n* = 4528), Atezolizumab (*n* = 2094), and Tislelizumab (*n* = 223). A summary of the hepatobiliary adverse effects related to each medication is presented in Table 1.

**TABLE 1:** Reported characteristics of hepatobiliary disorders AEs associated with ICIs drugs.

Characteristics	All immune checkpoint inhibitors drugs	Durvalumab	Pembrolizumab	Toripalimab	Nivolumab	Atezolizumab	Tislelizumab
Gender, *n* (%)							
Female	4093 (34.36%)	202 (25.31%)	1860 (43.94%)	—	1454 (32.11%)	577 (27.55%)	—
Male	6352 (53.32%)	392 (49.12%)	2236 (52.82%)	2 (5.40%)	2644 (58.39%)	1078 (51.48%)	—
Unknown	1468 (0.01%)	204 (25.56%)	137 (3.24%)	35 (94.59%)	430 (0.02%)	439 (20.96%)	223 (100%)
Weight (kg), *n* (%)							
< 80	3083 (25.88%)	238 (29.82%)	1081 (25.54%)	2 (5.40%)	1146 (25.31%)	616 (29.42%)	—
80–100	695 (5.83%)	40 (5.01%)	193 (4.56%)	—	318 (7.02%)	144 (6.88%)	—
> 100	207 (1.74%)	4 (0.50%)	62 (1.46%)	—	110 (2.43%)	31 (1.48%)	—
Unknown	7928 (66.55%)	516 (64.66%)	2897 (68.44%)	35 (94.59%)	2954 (65.24%)	1303 (62.22%)	223 (100%)
Median (kg)	64.9	61	63	60.75	67	64.4	—
Age (years), *n* (%)							
< 18	23 (0.19%)	1 (0.12%)	6 (0.14%)	—	15 (0.33%)	1 (0.05%)	—
18–44	644 (5.40%)	16 (2.00%)	234 (5.53%)	—	313 (6.91%)	81 (3.87%)	—
44–65	3663 (30.75%)	214 (26.82%)	1336 (31.56%)	—	1547 (34.16%)	566 (27.03%)	—
> 65	5174 (43.43%)	385 (48.24%)	2007 (47.41%)	2 (5.40%)	1887 (41.67%)	893 (42.64%)	—
Unknown	2409 (20.22%)	182 (22.81%)	650 (15.36%)	35 (94.59%)	766 (16.92%)	553 (26.41%)	223 (100%)
Median (years)	67	69	68	71	66	68	—
Occupation of reporters, *n* (%)							
Consumer (CN)	1631 (13.69%)	58 (7.27%)	911 (21.52%)	1 (2.70%)	558 (12.32%)	101 (4.82%)	2 (0.90%)
Physician (MD)	6853 (57.53%)	619 (77.57%)	2365 (55.87%)	7 (18.92%)	2242 (49.51%)	1611 (76.93%)	9 (4.04%)
Pharmacist (PH)	758 (6.36%)	20 (2.51%)	340 (8.03%)	5 (13.51%)	297 (6.56%)	94 (4.49%)	2 (0.90%)
Lawyer (LW)	5 (0.04%)	—	2 (0.05%)	—	3 (0.07%)	—	—
Other health‐professional (OT)	2579 (21.65%)	42 (5.26%)	602 (14.22%)	24 (64.86%)	1421 (31.38%)	280 (13.37%)	210 (94.17%)
Unknown	87 (0.73%)	59 (7.39%)	13 (0.31%)	—	7 (0.15%)	8 (0.38%)	—
Outcomes, *n* (%)							
Death (DE)	2451 (20.57%)	143 (17.92%)	769 (18.17%)	2 (5.40%)	1066 (23.54%)	469 (22.40%)	2 (0.90%)
Disability (DS)	139 (1.17%)	12 (1.50%)	54 (1.28%)	1 (2.70%)	37 (0.82%)	22 (1.05%)	13 (5.83%)
Hospitalization (HO)	4352 (36.53%)	246 (30.83%)	1658 (39.17%)	18 (48.65%)	1617 (35.71%)	681 (32.52%)	132 (59.19%)
Lifc‐threatening (LT)	673 (5.65%)	80 (10.02%)	234 (5.53%)	2 (5.40%)	283 (6.25%)	66 (3.15%)	8 (3.59%)
Other serious (OT)	4142 (34.77%)	305 (38.22%)	1457 (34.42%)	14 (37.84%)	1465 (32.35%)	833 (39.78%)	68 (30.49%)
Required intervention to prevent permanent impairment/damage (RI)	7 (0.06%)	—	2 (0.05%)	—	5 (0.11%)	—	—
Congenital anomaly (CA)	3 (0.02%)	1 (0.12%)	—	—	2 (0.04%)	—	—
Unknown	146 (1.22%)	11 (1.38%)	59 (1.39%)	—	53 (1.17%)	23 (1.10%)	—
Indication, *n* (%)							
Non‐small cell lung cancer	1351 (11.67%)	201 (27.50%)	457 (11.46%)	—	396 (8.77%)	297 (14.24%)	—
Malignant melanoma	1120 (9.67%)	—	198 (4.97%)	3 (8.11%)	899 (19.91%)	20 (0.96%)	—
Metastatic malignant melanoma	516 (4.46%)	—	150 (3.76%)	—	358 (7.93%)	8 (0.38%)	—
Triple negative breast cancer	404 (3.49%)	3 (0.41%)	320 (8.03%)	—	2 (0.04%)	79 (3.79%)	—
Lung adenocarcinoma	385 (3.33%)	9 (1.23%)	215 (5.39%)	—	66 (1.46%)	83 (3.98%)	12 (5.38%)
Hepatocellular carcinoma	1134 (9.80%)	182 (24.90%)	13 (0.33%)	—	122 (2.70%)	815 (39.07%)	2 (0.90%)
Bile duct cancer	133 (1.15%)	107 (14.64%)	15 (0.38%)	1 (2.70%)	4 (0.09%)	5 (0.24%)	1 (0.45%)
Others	6534 (56.44%)	229 (31.33%)	2619 (65.69%)	33 (89.19%)	2668 (59.09%)	777 (37.25%)	208 (93.27%)

Male patients experienced a higher overall incidence of hepatobiliary AEs (*n* = 6352; 53.32%) compared to female patients (*n* = 4093; 34.36%). A higher proportion of cases occurred in individuals weighing less than 80 kg (*n* = 3083; 25.88%), with a median weight of 64.9 kg. Additionally, patients older than 65 years accounted for a greater proportion of cases (*n* = 5174; 43.43%), with an average age of 67 years.

**FIGURE 2:**
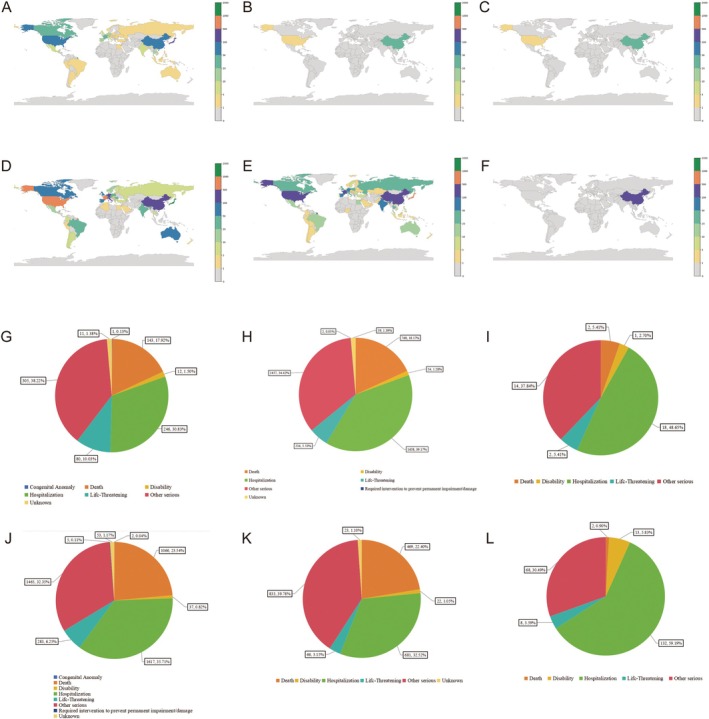
Geographic distribution and clinical outcomes of hepatobiliary AEs associated with ICIs. (A–F) World maps showing the country‐level frequency of hepatobiliary adverse event reports for: (A) Durvalumab, (B) Pembrolizumab, (C) Toripalimab, (D) Nivolumab, (E) Atezolizumab, and (F) Tislelizumab. (G–L) Proportional distribution of clinical outcomes (e.g., hospitalization, death) associated with hepatobiliary AEs for each drug: (G) Durvalumab, (H) Pembrolizumab, (I) Toripalimab, (J) Nivolumab, (K) Atezolizumab, and (L) Tislelizumab.

Physicians submitted a large portion of AEs reports (6853; 57.53%), followed by consumers (1631; 13.69%). Reports for Durvalumab, Toripalimab, and Tislelizumab primarily originated from China, whereas those for Pembrolizumab were largely submitted from Japan. Reports for Atezolizumab originated from China and the United States, whereas Nivolumab reports predominantly came from the United States and France (see Figure 2A–F).

As shown in Figure 2G–L, the most common outcomes associated with hepatobiliary AEs were hospitalization (*n* = 4352; 36.53%) and death (*n* = 2451; 20.57%). ICIs were primarily administered for non‐small cell lung cancer (*n* = 1351; 11.67%), and to a lesser extent for lung adenocarcinoma (*n* = 385; 3.33%) and cholangiocarcinoma (*n* = 133; 1.15%).

### 3.2 Signals Associated With Different Immune Checkpoint Inhibitor Drugs

All six ICIs analyzed in this study demonstrated positive signals for hepatobiliary system disorder AEs, as summarized in Table 2. The RORs with 95% CIs for each drug were: Durvalumab 4.28 (CI: 4.01–4.58), Pembrolizumab 3.93 (CI: 3.82–4.05), Toripalimab 3.73 (CI: 2.70–5.16), Nivolumab 3.42 (CI: 3.32–3.51), Atezolizumab 5.07 (CI: 4.87–5.28), and Tislelizumab 6.94 (CI: 6.07–7.93). The results show a statistically significant link between each of these medications and hepatobiliary AEs in the FAERS database.

**TABLE 2:** Signal detection for ICIs drug‐associated hepatobiliary disorders AEs.

Immune checkpoint inhibitors	The report number	ROR (95% CL)	PRR (chi^2^)	IC (IC025)	EBGM (EBGM05)
Durvalumab	798	4.28 (4.01, 4.58)	4.16 (2195.54)	2.05 (1.95)	4.15 (3.88)
Pembrolizumab	4233	3.93 (3.82, 4.05)	3.83 (10 386.14)	1.93 (1.88)	3.8 (3.7)
Toripalimab	37	3.73 (2.7, 5.16)	3.64 (73.43)	1.86 (1.3)	3.64 (2.63)
Nivolumab	4528	3.42 (3.32, 3.51)	3.34 (8598.7)	1.73 (1.69)	3.32 (3.23)
Atezolizumab	2094	5.07 (4.87, 5.28)	4.89 (7568.98)	2.28 (2.22)	4.87 (4.68)
Tislelizumab	223	6.94 (6.07, 7.93)	6.58 (1098.01)	2.72 (2.49)	6.58 (5.76)

**FIGURE 3:**
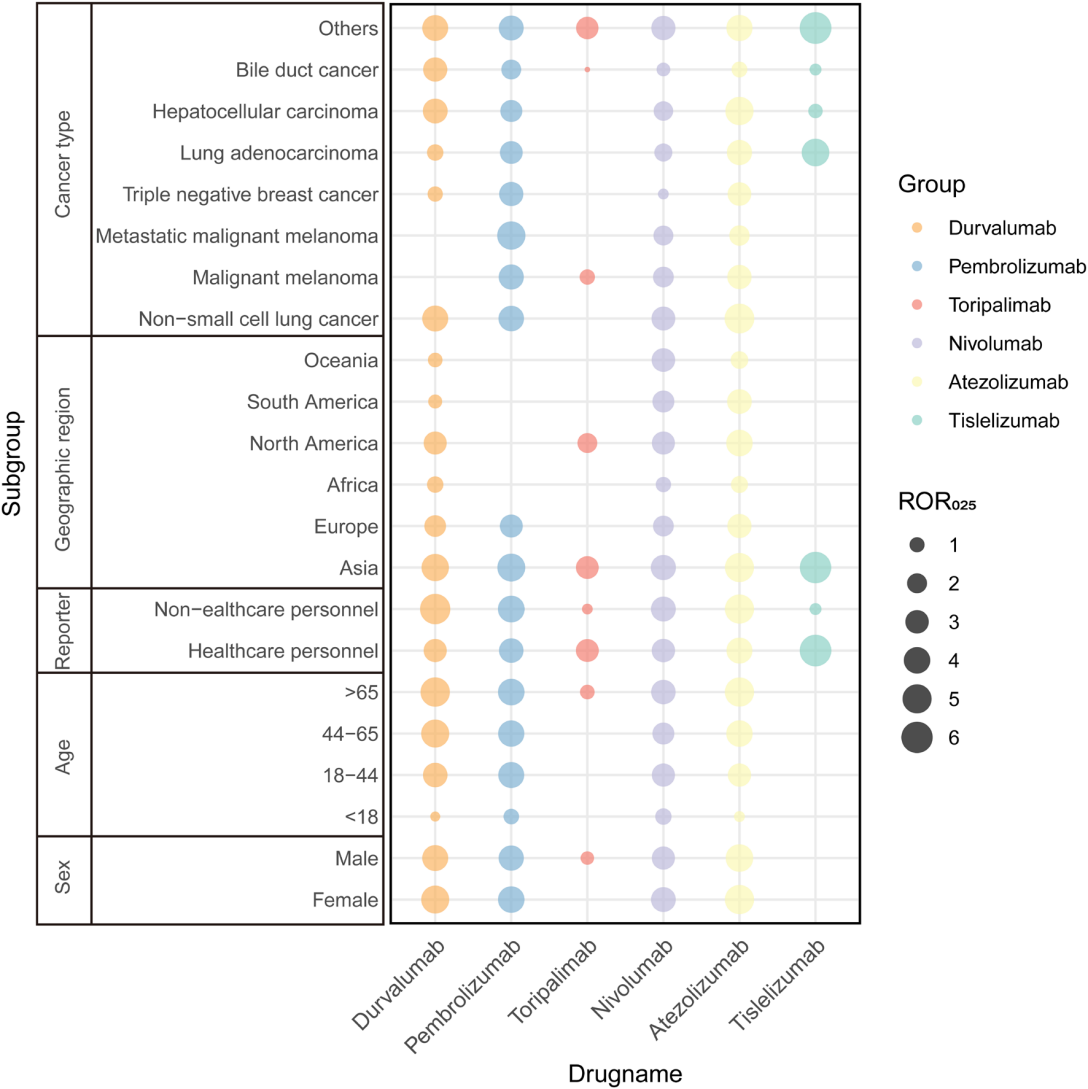
Signal intensity of ICIs versus hepatobiliary AEs by sex, age group, reporter, geographic region, and cancer type.

To minimize the influence of confounding factors on ICI‐related hepatobiliary AEs, we conducted a multivariate logistic regression analysis that adjusted for age, sex, weight, reporter type, reporting region, tumor type, and pre‐existing liver conditions. aRORs and their 95% confidence CIs were calculated. Additionally, to reduce potential distortion from concomitant medications that may contribute to hepatobiliary AEs, we listed the top 50 most frequently co‐administered drugs in Table S1. Based on FDA labeling information, several of these drugs—such as carboplatin, acetaminophen, axitinib, and cabozantinib—were identified as potentially associated with hepatobiliary toxicity. These suspect drugs, along with other relevant variables, were incorporated into the logistic regression model as confounding covariates. The results aligned with those of the primary analysis, confirming that all six ICIs remained significantly associated with hepatobiliary‐related AEs (Table 3). Furthermore, subgroup analyses revealed that the safety signals were largely consistent across all predefined subgroups with adequate sample sizes (Figure 3).

**TABLE 3:** Adjusted reporting odds ratio (aROR) for ICIs associated with hepatobiliary‐related AEs compared to other treatments in the FAERS database.

Drugs	*β*	SE	*Z*	*p*	aROR (95% CI)^a^
Durvalumab	1.200	0.040	30.512	< 0.001	3.39 (3.13, 3.66)
Pembrolizumab	1.037	0.016	64.812	< 0.001	2.82 (2.73, 2.91)
Toripalimab	0.917	0.166	5.529	< 0.001	2.50 (1.81, 3.46)
Nivolumab	0.972	0.015	64.807	< 0.001	2.64 (2.57, 2.72)
Atezolizumab	1.413	0.021	67.818	< 0.001	4.11 (3.94, 4.28)
Tislelizumab	1.689	0.070	24.107	< 0.001	5.41 (4.72, 6.21)

*Note:* For variables with 100% missing values in some ICIs, these variables are excluded in the multivariate logistic regression to ensure the validity of the aROR.

^a^ The aROR has been adjusted for age, sex, weight, reporter type, reporting region, tumor type, pre‐existing liver conditions, and potential concomitant medications.

### 3.3 Characterization of Adverse Effects at the PT Level

**FIGURE 4:**
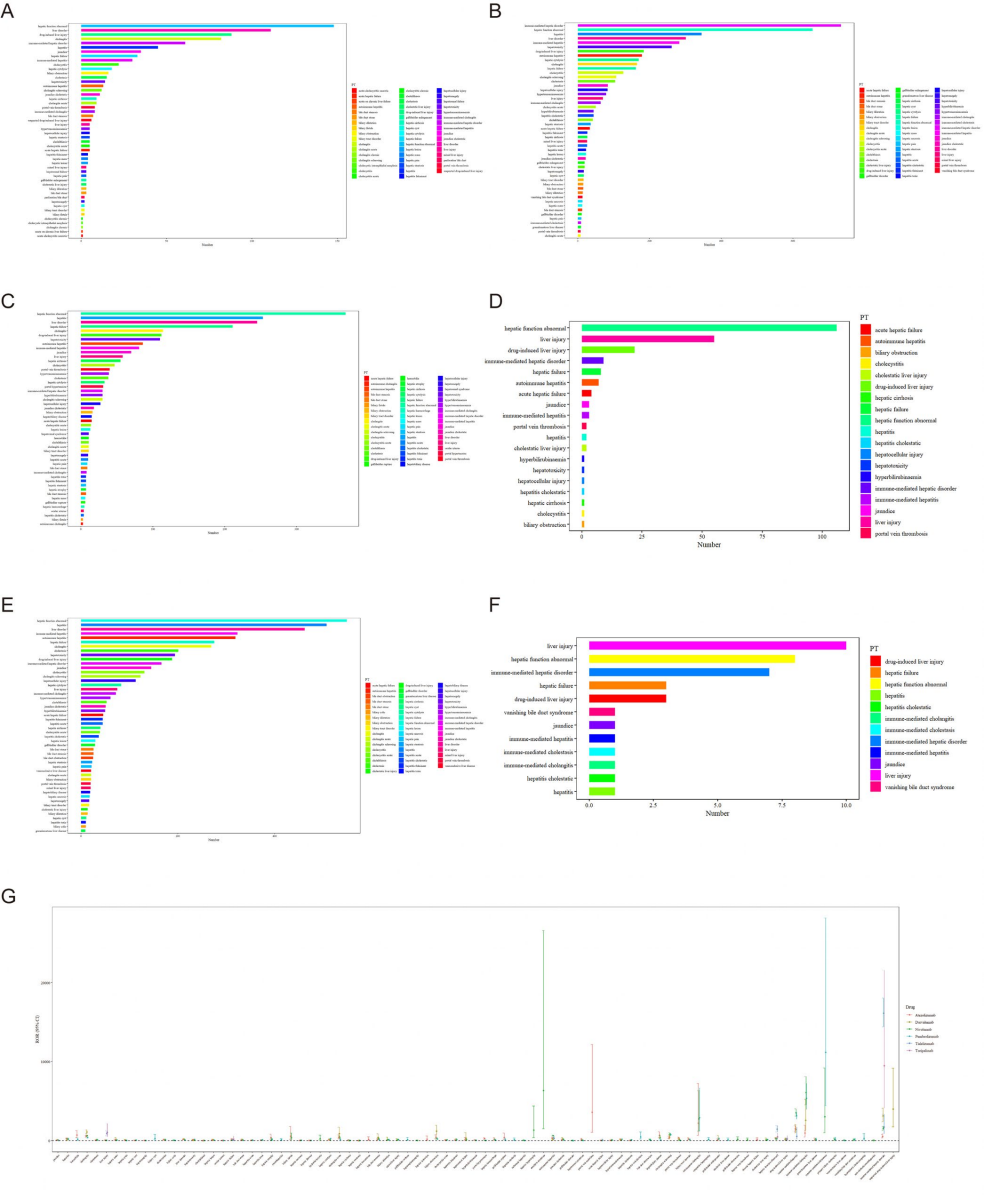
Common hepatobiliary AEs and disproportionality analysis by preferred term (PT) across ICIs. (A–F) Bar charts showing the most frequently reported hepatobiliary AEs (by PT level) associated with each ICI: (A) top 50 PTs for Durvalumab, (B) top 50 PTs for Pembrolizumab, (C) top 50 PTs for Atezolizumab, (D) top 19 PTs for Tislelizumab, (E) top 50 PTs for Nivolumab, and (F) top 12 PTs for Toripalimab. (G) Forest plot displaying the reporting odds ratios (RORs) for the top PT‐level hepatobiliary AEs across the six ICIs.

We analyzed AEs at the PT level for all six ICIs, each of which showed significant associations with hepatobiliary system disorders under the SOC. The top 50 PTs were identified for Durvalumab, Pembrolizumab, Nivolumab, and Atezolizumab, whereas 12 PTs were analyzed for Toripalimab and 19 PTs for Tislelizumab (Figure 4A–F).

The most frequently reported PTs were as follows: Durvalumab: immune‐mediated liver disease (*n* = 734), abnormal liver function (*n* = 655), hepatitis (*n* = 345), liver damage (*n* = 301). Pembrolizumab: abnormal liver function (*n* = 148), liver damage (*n* = 111), pharmacologic liver injury (*n* = 88), cholangitis (*n* = 82). Nivolumab: abnormal liver function (*n* = 549), hepatitis (*n* = 507), liver damage (*n* = 462), immune‐mediated liver disease (*n* = 323). Atezolizumab: abnormal liver function (*n* = 368), hepatitis (*n* = 253), liver damage (*n* = 245), liver failure (*n* = 211). Toripalimab: liver injury (*n* = 10), liver function abnormalities (*n* = 8), immune‐mediated liver disease (*n* = 7), drug‐mediated liver injury (*n* = 3), and Tislelizumab: liver function abnormalities (*n* = 106), liver injury (*n* = 55), drug‐mediated liver injury (*n* = 22), immune‐mediated liver disease (*n* = 9).

Abnormal liver function was the most common life‐threatening or fatal adverse event among the top 10 PTs for patients treated with Pembrolizumab, Nivolumab, Atezolizumab, and Tislelizumab. In contrast, immune‐mediated liver disease was most common in Durvalumab‐treated patients, and liver injury was the leading PT in the Toripalimab group (Tables S2–S7).

Disproportionality analysis revealed the following number of positive PT‐level signals: Durvalumab: 40 out of 75 PTs, Pembrolizumab: 73 out of 110 PTs, Toripalimab: 5 out of 12 PTs, Nivolumab: 71 out of 105 PTs, Atezolizumab: 60 out of 90 PTs, and Tislelizumab: 9 out of 19 PTs (Figure 4G).

Logistic regression analysis (Table 4) showed that age, body weight, gender, and the top three clinical indications (based on report frequency) did not have a meaningful statistical effect (*p* > 0.05) regarding the risk of hepatobiliary disorders associated with any of the ICIs studied.

**TABLE 4:** Logistic regression results of Hepatobiliary disorders ADEs across different factors.

Factors	*p*	Exp(*B*)	95% confidence interval of Exp(*B*)	
			Lower limit	Upper limit
Durvalumab				
Age	0.835	0.990	0.897	1.092
Body weight	0.542	1.024	0.949	1.104
Sex	0.278	3.538	0.361	34.700
Non‐small cell lung cancer	0.234	4.201	0.395	44.719
Hepatocellular carcinoma	0.701	1.601	0.145	17.703
Bile duct cancer	0.242	0.316	0.046	2.175
Atezolizumab				
Age	0.182	0.961	0.906	1.019
Body weight	0.517	1.013	0.974	1.054
Sex	0.432	1.688	0.457	6.237
Hepatocellular carcinoma	0.501	1.639	0.389	6.902
Non‐small cell lung cancer	0.638	0.707	0.167	2.995
Lung adenocarcinoma	0.997	39 597 229.265	0.000	
Nivolumab				
Age	0.349	1.026	0.972	1.083
Body weight	0.607	1.012	0.966	1.060
Sex	0.415	0.502	0.096	2.636
Malignant melanoma	0.994	10 608 834.802	0.000	
Non‐small cell lung cancer	/	/	/	/
Metastatic malignant melanoma	0.996	10 334 642.779	0.000	
Pembrolizumab				
Age	0.669	1.016	0.944	1.094
Body weight	0.632	1.015	0.956	1.077
Sex	0.137	13.604	0.438	422.680
Non‐small cell lung cancer	0.996	8 730 838.809	0.000	
Triple negative breast cancer	0.052	31.442	0.965	1024.254
Lung adenocarcinoma	0.997	5 827 826.771	0.000	

### 3.4 The Analysis of the Time of Onset

We analyzed the onset times of 15 hepatobiliary AEs associated with Durvalumab, Pembrolizumab, Nivolumab, and Atezolizumab; 9 events associated with Tislelizumab; and 4 events related to Toripalimab.

**FIGURE 5:**
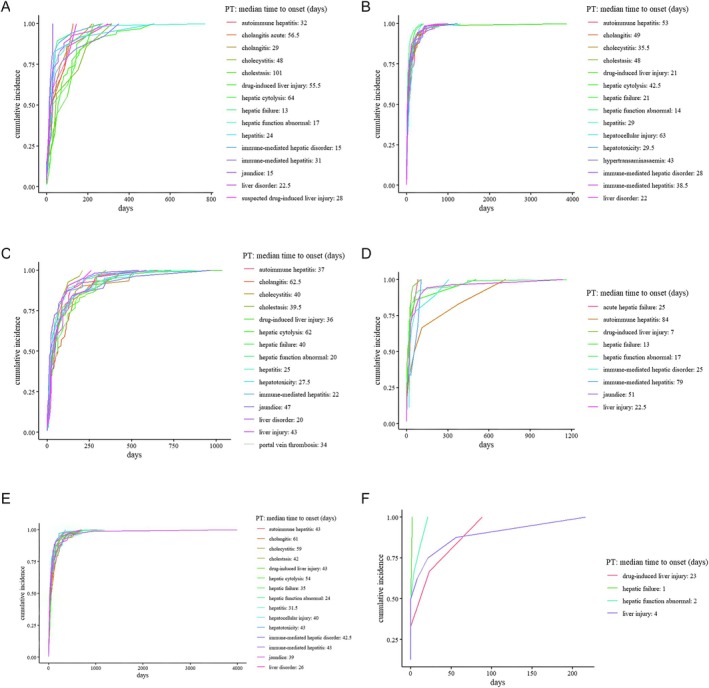
Time‐to‐onset analysis of hepatobiliary AEs associated with ICIs. Kaplan–Meier–style plots or distribution curves illustrating the time to occurrence of hepatobiliary AEs for each drug: (A) Durvalumab, (B) Pembrolizumab, (C) Atezolizumab, (D) Tislelizumab, (E) Nivolumab, and (F) Toripalimab.

Among these, the longest median time to onset was observed for: Cholestasis in the Durvalumab group: 101 days, Hepatocellular injury in the Pembrolizumab group: 63 days, Cholangitis in the Nivolumab group: 61 days, Cholangitis in the Atezolizumab group: 62.5 days, Autoimmune hepatitis in the Tislelizumab group: 84 days, Pharmacologic liver injury in the Toripalimab group: 23 days, and other PT‐specific onset times are shown in Figure 5.

Goodness‐of‐fit results (Table S8) indicated that the Weibull distribution adequately characterized the TTO of hepatobiliary AEs across all ICIs. All six agents were identified as exhibiting an early failure pattern (*β* < 1), indicating that hepatobiliary AEs tend to occur shortly after the initiation of treatment for several ICIs. Detailed parameters from the Weibull analysis are presented in Table 5.

**TABLE 5:** Time to onset of ICIs drugs related hepatobiliary disorders AEs and Weibull distribution analysis.

Drug	Time to onset (days)		Weibull distribution		
	Case reports	Median (IQR)	Scale parameter: *α* (95% CI)	Shape parameter: *β* (95% CI)	Type
Durvalumab	339	28 (13.5–66.5)	57.01 (48.96–65.07)	0.81 (0.74–0.87)	Early failure
Pembrolizumab	1720	29 (10–84.25)	70.34 (65.33–75.35)	0.73 (0.70–0.75)	Early failure
Toripalimab	22	3 (0–21)	37.96 (10.95–64.97)	0.84 (0.49–1.19)	Early failure
Nivolumab	2314	39 (14–98)	80.31 (75.68–84.94)	0.76 (0.74–0.79)	Early failure
Atezolizumab	1021	31 (12–103)	73.29 (66.63–79.95)	0.72 (0.69–0.76)	Early failure
Tislelizumab	207	20 (5.5–35)	40.30 (30.88–49.73)	0.63 (0.57–0.69)	Early failure

## 4 Discussion

This investigation methodically analyzed the hepatobiliary toxicity of six ICIs that are utilized in clinical treatments—Durvalumab, Atezolizumab, Nivolumab, Pembrolizumab, Toripalimab, and Tislelizumab—utilizing actual data from the FAERS. A comprehensive signal detection approach was employed, integrating the PRR method with the BCPNN model. In addition to adverse event characterization under the hepatobiliary SOC, multifactorial logistic regression and TTO analyses were performed. Despite the inherent limitations of this retrospective study, which do not allow for definitive establishment of causality between ICIs and hepatobiliary AEs, the findings remain significant and provide novel, evidence‐based insights for clinical practice.

ICIs have shown notable survival benefits over traditional chemotherapy, especially in patients with elevated PD‐L1 expression, while combination strategies have enhanced progression‐free survival (PFS) and level of well‐being [6, 7, 8, 9]. Despite a lower overall incidence of irAEs compared to cytotoxic agents like platinum compounds, the potential for organ‐specific toxicity, especially hepatobiliary toxicity, necessitates systematic evaluation [10]. Real‐world safety data remain limited, which underscores the importance of large‐scale pharmacovigilance assessments like this study.

Our findings highlight substantial heterogeneity in hepatobiliary toxicity risk among different ICI drugs. For example, Pembrolizumab was associated with 73 positive signals out of 110 prespecified PTs, and Nivolumab with 71 out of 105, whereas Tislelizumab showed only 9 out of 19. These discrepancies suggest that intrinsic drug properties are a key factor in toxicity risk. Logistic regression confirmed that demographic variables and indication patterns were not significantly associated with hepatobiliary AE risk, further reinforcing the influence of drug‐specific effects.

A key innovation of this study lies in the detailed profiling of hepatobiliary toxicity for each ICI based on real‐world data. Time‐series analysis revealed significant differences in onset times between drugs. Toripalimab had the earliest median onset of liver injury (23 days), indicating a potential for acute toxicity shortly after treatment initiation. This suggests that pre‐treatment risk stratification and early monitoring protocols should be implemented—particularly for high‐risk agents like Toripalimab—to achieve a balance between therapeutic efficacy and organ protection.

We also found that male patients experienced a higher proportion of hepatobiliary AEs, potentially linked to androgen‐induced PD‐L1 expression in cholangiocytes. This observation is consistent with findings from the CheckMate 040 trial [11]. The increased occurrence of AEs in older patients might be due to immunosenescence and reduced T‐cell function [12].

Our subgroup analysis identified additional mechanistic differences between PD‐1 and PD‐L1 inhibitors. Nivolumab was more often associated with hepatitis and immune‐mediated liver disease, while Pembrolizumab had a higher incidence of drug‐induced liver injury and cholangitis. Previous studies have indicated that PD‐1 inhibitor‐associated hepatotoxicity primarily manifests as hepatitis, with a risk 5.85 times higher than the chemotherapy group for all grades and 5.66 times higher for high grades [13]. These variations might indicate a disruption in immune tolerance upheld by liver‐resident cells, such as LSECs, Kupffer cells, and dendritic cells, along with changes in MHC expression [1]. Among PD‐L1 inhibitors, immune‐mediated liver disease and hepatitis predominated in Durvalumab cases, while liver failure was more commonly linked to Atezolizumab. This could indicate the pattern of PD‐L1 expression in liver tissues, particularly under inflammatory conditions [14].

Our findings, which suggest distinct hepatotoxicity profiles between PD‐1 and PD‐L1 inhibitors, are supported by emerging mechanistic insights into the differential roles of these immune checkpoints within the hepatic microenvironment. PD‐1 inhibitors primarily work by blocking the interaction between PD‐1 and PD‐L1, leading to excessive activation of T cells and consequently causing hepatocyte damage [15, 16]. PD‐L1 inhibitors (such as Atezolizumab) may not only block the PD‐1/PD‐L1 pathway but also induce necroptosis by directly acting on PD‐L1‐expressing hepatocytes [17]. In vitro studies indicate that Atezolizumab induces hepatocyte death by activating the phosphorylation pathways of RIP3 and MLKL proteins [18]. Furthermore, liver tissue following PD‐1 inhibitor treatment exhibits significantly increased PD‐1+ lymphocyte infiltration, whereas PD‐L1 inhibitors are more likely to cause cholestatic injury [19, 20]. Moreover, the incidence of hepatotoxicity with PD‐1 inhibitors (such as Nivolumab and Pembrolizumab) is significantly higher than that with PD‐L1 inhibitors (such as Atezolizumab) in clinical practice. A meta‐analysis of 117 clinical trials revealed incidence rates of elevated ALT and AST in the PD‐1 inhibitor group were 6.01% and 6.84%, respectively, compared to only 3.60% and 3.72% in the PD‐L1 inhibitor group [20]. Additionally, the incidence of high‐grade (≥ Grade 3) hepatotoxicity was significantly higher with PD‐1 inhibitors (1.54% vs. 1.03%) [20]. This difference may be related to the stronger direct T‐cell activation effect of PD‐1 inhibitors [15, 16].

Additionally, Toripalimab and Tislelizumab, both domestically developed PD‐1 inhibitors, were associated with fewer hepatobiliary AEs reports. This could be due to limited exposure and smaller sample sizes in real‐world settings rather than inherently lower toxicity. Notably, Tislelizumab had a relatively high ROR (6.94; 95% CI: 6.07–7.93), suggesting that signal strength may increase with broader clinical use.

Management of irAEs remains a critical challenge. Current guidelines recommend liver function assessment and viral hepatitis screening before initiating ICI therapy [21]. For mild‐to‐moderate irAEs, temporary discontinuation until resolution is advised, while severe irAEs warrant permanent cessation of therapy [22]. There is increasing recognition that irAEs may correlate with improved oncologic outcomes (e.g., PFS, ORR, OS) in patients with manageable toxicities [23]. However, corticosteroid use prior to ICI initiation has been associated with worse survival outcomes [24], highlighting the need for updated, evidence‐based guidelines on immunosuppressive therapy in this context. Tocilizumab has emerged as a possible treatment for steroid‐refractory irAEs, though randomized trials are required to validate its efficacy and safety [25].

This study also utilized the Weibull distribution to characterize TTO patterns and propose a tailored pharmacovigilance strategy. Cholestasis was the latest‐onset AEs observed (Durvalumab group, ~101 days), requiring extended monitoring. Hepatocellular injury occurred around 63 days after starting Pembrolizumab, and cholangitis presented within 60–63 days for both Nivolumab and Atezolizumab. Autoimmune hepatitis was noted around 84 days with Tislelizumab, and pharmacologic liver injury emerged as early as 23 days with Toripalimab. These results highlight the importance of creating adaptable, drug‐specific monitoring strategies within the initial 3 months of therapy. Biomarker‐driven strategies may further optimize adverse event prediction and management in the future.

This study has several innovative aspects. First, it represents the first disproportionality analysis of ICI‐associated hepatobiliary AEs using a pharmacovigilance database, thereby providing a large sample size. Second, multiple disproportionality methods and sensitivity analyses were employed to initially explore and validate the signals. Furthermore, TTO analysis was applied to compare latency differences among various ICIs. However, the study also has several limitations. The pharmacovigilance database has inherent constraints, including underreporting, variability in reporting enthusiasm (e.g., intensified surveillance after drug approval), and insufficient coverage of delayed AEs. These may distort disproportionality signals (overestimation or underestimation) and impede the detection of delayed events. Moreover, the retrospective design limits the inference of causality. Although we adjusted for confounders such as age, sex, weight, reporter type, region, tumor type, pre‐existing liver conditions, and concomitant medications, inherent reporting biases and incomplete data may still introduce residual bias. Importantly, due to complex drug combinations and incomplete co‐medication records, the risk of confounding by concomitant prescriptions could not be fully eliminated. Additionally, the lack of key clinical details—such as tumor stage, treatment lines, metastasis status, and drug dosage—may result in unmeasured confounding, potentially affecting the accuracy of the results. Therefore, prospective cohort studies are warranted to establish causal relationships and validate these findings. Moreover, the predominance of Western data limits generalizability to other populations. Broader international reporting and harmonization are needed for global pharmacovigilance improvements [26, 27].

## 5 Conclusion

This study utilized real‐world data from the FAERS database to assess the hepatobiliary safety profiles of six ICIs used in cancer treatment: Durvalumab, Atezolizumab, Nivolumab, Pembrolizumab, Toripalimab, and Tislelizumab. Among these, Atezolizumab and Tislelizumab exhibited stronger signals for hepatobiliary system disorders at the SOC level compared to other agents.

The study identified distinct mechanisms of hepatotoxicity between PD‐1 and PD‐L1 inhibitors. PD‐1 inhibitors (e.g., Nivolumab, Pembrolizumab) were primarily associated with hepatitis and liver function abnormalities, whereas PD‐L1 inhibitors (e.g., Durvalumab, Atezolizumab) were more frequently linked to immune‐mediated liver disease and liver failure. These variations are probably due to differing hepatic expression patterns and the immunoregulatory functions of PD‐1 and PD‐L1 in the liver microenvironment.

To ensure the safe and effective use of ICIs in clinical practice, future studies should prioritize the optimization of irAE management through multidisciplinary collaboration and prospective clinical trials. Additionally, expanded validation studies are essential to address the limitations of passive pharmacovigilance data from FAERS, including underreporting and regional bias.

## Author Contributions

T.Z. and F.Q. collaborated on the article's conception and creativity. Under T.Z.'s guidance, Y.C., Y.Z., and K.Z. conducted the data download and analysis. Y.C. and K.Z. drafted the initial manuscript, whereas Y.Z. handled the literature retrieval. Y.C., Y.Z., N.L., and Y.Z. were responsible for drawing and table creation. F.Q., T.Z., and Y.Z. revised the article. All authors have read and approved the final manuscript.

## Ethics Statement

Considering that the FAERS database is publicly accessible, and patient records are anonymous and de‐identified, it does not involve informed consent or ethical approval.

## Conflicts of Interest

The authors declare no conflicts of interest.

## Supporting information


**Table S1:** Top 50 concomitant medications in patients with ICI‐associated hepatobiliary AEs in the FAERS database.
**Table S2:** Detailed breakdown of hepatobiliary adverse events associated with Durvalumab. This table presents all Preferred Terms (PTs) related to hepatobiliary events reported for Durvalumab in the FAERS database. The table includes counts for various outcomes, including death (DE), hospitalization (HO), and life‐threatening events (LT), among others.
**Table S3:** Detailed breakdown of hepatobiliary adverse events associated with Pembrolizumab. This table lists all PT‐level hepatobiliary events reported for Pembrolizumab, including frequencies and corresponding clinical outcomes (e.g., DE, HO, DS).
**Table S4:** Detailed breakdown of hepatobiliary adverse events associated with Toripalimab. PT‐level adverse events for Toripalimab are shown with associated outcome counts, enabling risk stratification for liver‐related complications.
**Table S5:** Detailed breakdown of hepatobiliary adverse events associated with Nivolumab. The table provides a comprehensive view of liver‐related AEs for Nivolumab users, organized by PT and outcome type.
**Table S6:** Detailed breakdown of hepatobiliary adverse events associated with Atezolizumab. Includes PT‐level reporting of liver toxicities for Atezolizumab and the respective frequencies of adverse clinical outcomes.
**Table S7:** Detailed breakdown of hepatobiliary adverse events associated with Tislelizumab. Summarizes FAERS‐reported liver AEs for Tislelizumab by PT, including hospitalization, death, and other outcome classifications.
**Table S8:** Performance tests for goodness‐of‐fit of three parametric distribution models.

## Data Availability

Data sharing not applicable to this article as no datasets were generated or analysed during the current study.
